# Characterization of the complete mitochondrial genome sequence of *Nibea diacanthus* and its phylogenetic implication

**DOI:** 10.1080/23802359.2019.1698981

**Published:** 2020-01-14

**Authors:** Zehui Hu, Xuejun Chai, Yuebin Wang

**Affiliations:** Key Laboratory of Mariculture and Enhancement of Zhejiang Province, Marine Fisheries Research Institute of Zhejiang, Zhoushan, P.R. China

**Keywords:** *Nibea diacanthus*, mitochondrial genome, phylogenetic analysis, Sciaenidae

## Abstract

The blackspotted croaker (*Nibea diacanthus*) is an important food fish of Indo-West Pacific and China. To study the phylogenetic status, we sequenced the complete mitochondrial genome of *N. diacanthus*. The mitogenome is 16,532 bp in length and composed of 13 protein-coding genes, two rRNAs, 22 tRNAs, and a control region. The gene composition and the structural arrangement of *N. diacanthus* complete mtDNA were identical to most of other vertebrates. The phylogenetic analysis using the complete mitochondrial genome revealed that the *N. diacanthus* might be separated from *Nibea* genera of Argyrosominae, which was inconsistent with that based on morphology. The complete mitogenome data would be useful for the evolution and conservation genetic studies of Sciaenidae.

The blackspotted croaker *Nibea diacanthus* (Perciformes, Sciaenidae) is a kind of offshore warm-water bottom fish with obvious seasonal migration. It is widely distributed in China, North Korea, Japan, India, Ceylon, and Myanmar. It feeds on crustaceans and small fishes. The *N. diacanthus* grows faster, which the individual can reach more than 10 kg. It was regarded as the new fine species for offshore cage culture in China with good development prospects. The wild stocks of *N. diacanthus* have been collapsed due to overfishing and habitat degradation (Cheng and Zhang 1987). Therefore, knowledge about the complete mitogenome is vital to study the present population structure, genetic diversity, dispersal form, breeding pattern and effective conservation strategies. Hence, in the present study, we described the complete mitogenome of *N. diacanthus*.

Up to now, phylogenetic relationship of *N. diacanthus* was inconsistent with different researches. *Nibea diacanthus* was classified into the *Nibea* of Argyrosominae (Zhu et al. 1963), whereas Chen (2007) found that it should be separated from *Nibea* using mtDNA 16S rRNA. Researches of single gene or taxonomic might lose some significant evolutionary characters. Hence, this study expects to contribute to the phyogenetic analysis of the Sciaenidae and natural resources conservation of *N. diacanthus.*

In this study, the specimen of *N. diacanthus* captured from the coast of Raoping, Guangdong, located at 117°2′6″E, 23°31′54″N, and was stored in the fish specimen room of Zhejiang Marine Fisheries Research Institute (accession number: 20160715ND01). Caudal fins were stored in 95% ethanol at −20 °C. Genomic DNA were isolated using the high-salt procedure (Aljanabi and Martinez 1997). PCR primers were initially designed according to *N. albiflora* (HQ890947), *N. japonica* (KT184692), and *N. coibor* (KM233452). Subsequently, based on the received sequences, some additional primers were designed to supplement residual gaps. Finally, ContigExpress software was used to sequence analysis and assembly. The assembled mitochondrial genome was annotated by MitoFish (Iwasaki et al. 2013). All tRNA genes were reappraised using tRNAscan-SE1.21 (Lowe and Eddy 1997), which was also used to characterize the anti-codons of all tRNAs. Simultaneously, we downloaded 18 complete mitochondrial genome of Sciaenidae, which were aligned by means of Clustal W using BioEdit (Hall 2004). The best-fit model to nucleotide substitution of these genomes was Jmodel test2 (Darriba et al. 2012), via Alkaike information criteria (AICc). Finally, the phylogenetic analysis of Maximum-Likelihood (ML) was performed using MEGA 7.0 (Kumar et al. 2016), and the number of bootstrap replicates is 1000.

The complete mitochondrial genome of blackspotted croaker *N. diacanthus* was 16,532 bp in length (KY117237), containing 13 protein-coding genes, two ribosomal RNA genes (12S rRNA and 16S rRNA), 22 transfer RNA (tRNA) genes, and a control region. All of them are encoded on the heavy strand except NADH dehydrogenase subunit 6 (ND6) and eight tRNA genes (Gln, Pro, Glu, Ser, Tyr, Cys, Asn, and Ala) on the light strand. The base composition was T (25.4%), C (31.1%), A (27.5%), and G (16.0%). Therefore, the overall content A + T with 52.9%, is slightly rich than the G + C content (47.1%), similar to the values in other teleosts (Accession ID：KM233452, HQ890947, JQ286004 and HM447240).

Thirteen protein-coding genes were initiated with the orthodox ATG. A different pattern of codon usage was found in stop codons: TAA for *ND1*, *ND4L*, *ND6*, *ATP8*, TAG for *ND5*, and AGA for *COI*. The remaining seven genes had incomplete stop codon: TA– for *ND2*, *COIII*, and T– for *COII*, *ATP6*, *ND3*, *ND4* and *Cyt b*, which were presumably completed by post transcriptional polyadenylation (Ojala et al. 1981). Three overlapping regions were founded in protein-coding genes: *ATP8* and *ATP6* (9 nucleotides), *ATP6* and *COIII* (two nucleotide), *ND4L* and *ND4* (seven nucleotides). The length of all tRNAs ranged from 66 to 75 bp and their anti-condons were consistent with other fish of Sciaenidae. The 12S and 16S rRNA of *N. diacanthus* were 946 bp and 1720 bp in length respectively. They were located between tRNA^Phe^ and tRNA^Leu^ (UUR), separating by tRNA^Val^. The CR determined between tRNA^Pro^ and tRNA^Phe^ was 838 bp in length. The A + T content of the CR was 64% (T 29.8%, C 22.0%, A 34.2%, G 14.0%), which was higher than the average value of the whole mitochondrial genome (52.9%) of *N. diacanthus*.

The best-fit model to nucleotide substitution of these genomes was HKY + G + I. Phylogenetic analysis revealed that the 19 Sciaenidae species in China were grouped in two clusters (Figure 1). Johninae was in the basal position, indicating they are most primitive groups among the family Sciaenidae, which were consistent with the previous conclusions based on morphology. Then, the Argyrosominae, Pseudosciaeninae, and Sciaeniae formed the sister group. Specially, *N. diacanthus*, *Pennahia argentata* and other eight fish firstly clustered into the Argyrosominae clade (Figure 1), being inconsistent with the previous reports (Cheng and Zhang 1987). Due to high bootstrap values support, phylogeny validated that *N. diacanthus* might be separated from *Nibea* genera of Argyrosominae, which was highly inconsistent with that based on morphology (Zhu et al. 1963).

**Figure 1.**
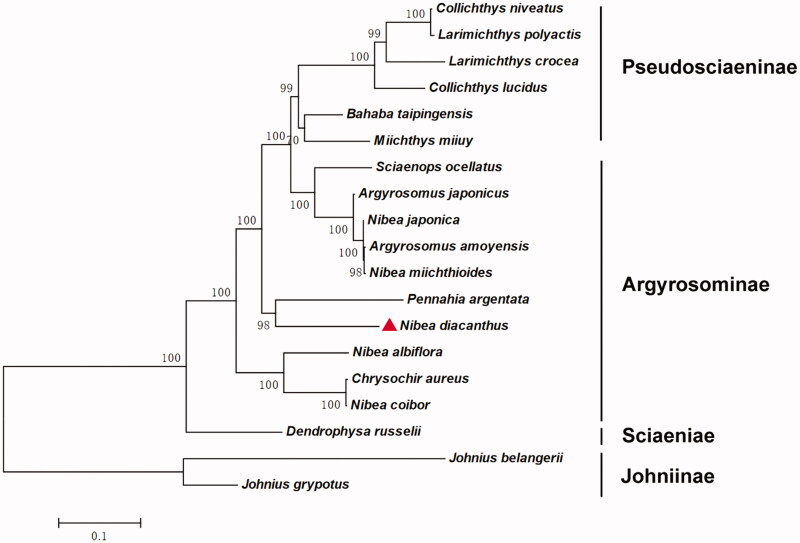
The phylogenetic relationship for fish of Sciaenidae. Note. 0.1 is the evolution scale of phylogenetic tree. GenBank Accession: *Argyrosomus japonicus* (JQ728563), *Argyrosomus amoyensis* (KM257863), *Bahaba taipingensis* (JX232404), *Chrysochir aureus* (JQ692068), *Collichthys niveatus* (HM219223), *Collichthys lucidus* (JN857362), *Dendrophysa russelii* (JQ728562), *Johnius belangerii* (KF211426), *Johnius grypotus* (KC491206), *Larimichthys polyactis* (GU586227), *Larimichthys crocea* (EU339149), *Miichthys miiuy* (HM447240), *Nibea miichthioides* (KU738606, in this study), *Nibea japonica* (KT184692), *Nibea albiflora* (HQ890947), *Nibea coibor* (KM233452), *Pennahia argentata* (KC545800), *Nibea diacanthus* (KY117237), and *Sciaenops ocellatus* (JQ286004).
